# Genetic Predictors for Sinusoidal Obstruction Syndrome—A Systematic Review

**DOI:** 10.3390/jpm11050347

**Published:** 2021-04-26

**Authors:** Nicolas Waespe, Sven Strebel, Simona Jurkovic Mlakar, Maja Krajinovic, Claudia Elisabeth Kuehni, Tiago Nava, Marc Ansari

**Affiliations:** 1 CANSEARCH Research Platform in Pediatric Oncology and Hematology, University of Geneva, 1205 Geneva, Switzerland; nicolas.waespe@ispm.unibe.ch (N.W.); sven.strebel@ispm.unibe.ch (S.S.); simona.mlakar@unige.ch (S.J.M.); tiago.nava@unige.ch (T.N.); 2 Institute of Social and Preventive Medicine, University of Bern, 3012 Bern, Switzerland; claudia.kuehni@ispm.unibe.ch; 3 Graduate School for Cellular and Biomedical Sciences (GCB), University of Bern, 3012 Bern, Switzerland; 4 Graduate School for Health Sciences (GHS), University of Bern, 3012 Bern, Switzerland; 5 Charles-Bruneau Cancer Center, CHU Sainte-Justine Research Center, Department of Pediatrics, Montreal, QC H3T 1C5, Canada; maja.krajinovic@umontreal.ca; 6 Clinical Pharmacology Unit, Department of Pediatrics, CHU Sainte-Justine, Montreal, QC H3T 1C5, Canada; 7 Department of Pharmacology, Faculty of Medicine, University of Montreal, Montreal, QC H3T 1J4, Canada; 8 Division of Pediatric Hematology/Oncology, Department of Pediatrics, Inselspital, Bern University Hospital, University of Bern, 3012 Bern, Switzerland; 9 Department of Women, Children and Adolescents, Division of Pediatric Oncology and Hematology, Geneva University Hospital, 1205 Geneva, Switzerland

**Keywords:** sinusoidal obstruction syndrome, genetic polymorphism, pharmacogenomic variants, genetic predisposition, genetic association studies, whole-exome sequencing, candidate gene analysis, hematopoietic stem cell transplantation, antineoplastic agents, systematic review

## Abstract

Sinusoidal obstruction syndrome (SOS) is a potentially life-threatening complication after hematopoietic stem cell transplantation (HSCT) or antineoplastic treatment without HSCT. Genetic variants were investigated for their association with SOS, but the evidence is inconclusive. We performed a systematic literature review to identify genes, gene variants, and methods of association analyses of genetic markers with SOS. We identified 23 studies after HSCT and 4 studies after antineoplastic treatment without HSCT. One study (4%) performed whole-exome sequencing (WES) and replicated the analysis in an independent cohort, 26 used a candidate-gene approach. Three studies included >200 participants (11%), and six were of high quality (22%). Variants in 34 genes were tested in candidate gene studies after HSCT. Variants in *GSTA1* were associated with SOS in three studies, *MTHFR* in two, and *CPS1*, *CTH*, *CYP2B6*, *GSTM1*, *GSTP1*, *HFE*, and *HPSE* in one study each. *UGT2B10* and *LNPK* variants were identified in a WES analysis. After exposure to antineoplastic agents without HSCT, variants in six genes were tested and only *GSTM1* was associated with SOS. There was a substantial heterogeneity of populations within and between studies. Future research should be based on sufficiently large homogenous samples, adjust for covariates, and replicate findings in independent cohorts.

## 1. Introduction

Sinusoidal obstruction syndrome (SOS) of the liver is a serious, potentially life-threatening complication occurring usually within the first 30 days after hematopoietic stem cell transplantation (HSCT) [1] or after treatment with some antineoplastic agents without HSCT [2]. Diagnostic criteria are based on clinical and laboratory findings including weight gain/ascites, hyperbilirubinemia, and hepatomegaly/right upper abdominal quadrant pain (Seattle criteria [3] and Baltimore criteria [4]). These were revised more recently to better reflect different forms of SOS presentation [5]. Depending on which criteria are used, the frequency of patients diagnosed with SOS might differ by two-fold [6]. Risk factors after HSCT include underlying disease (thalassemia major, leukemia, hemophagocytosis), pre-existing liver disease or injury, iron overload, laboratory markers (increased bilirubin and transaminases before HSCT), previous treatment with gemtuzumab ozogamicin, previous allogeneic HSCT, high-intensity conditioning regimens, and total body irradiation [5,7,8]. Children are twice as likely to develop SOS after HSCT than adults [9] and even higher within the first two years of life [5]. Antineoplastic agents associated with SOS without HSCT are alkylating agents, platinum agents (particularly oxaliplatin [10,11]), and purine analogues [12,13,14]. SOS was also reported after acute lymphoblastic leukemia (ALL) induction treatment [13] and treatment with actinomycin D for nephroblastoma [15]. 

The pathogenesis of SOS is complex and includes damage to endothelial cells and hepatocytes. Cytotoxic agents and their metabolites lead to the activation of stress response mechanisms, and loss of integrity of the endothelial lining in the liver sinusoidal space [16]. Cytokines released by the damaged tissues further enhance the damaging process and activate the coagulation cascade leading to thrombi in the liver microvasculature [17]. All of these processes result in sinusoidal obstruction, liver cell injury, and hepato-renal syndrome associated with kidney failure, and death [18]. Obstruction of the sinusoidal spaces was found to be secondary to endothelial damage with inflammation and locally activated coagulation with an increase in procoagulant factors and a decrease in antithrombotic proteins. Subsequently, venous outflow obstruction of the liver causes damage to the liver cells [19,20]. The molecular mechanisms thought to affect SOS include the cytochrome P-450 enzymatic system, which plays an important role in the clearance of toxic metabolites of chemotherapeutics (e.g., cyclophosphamide) and the glutathione pathway, which is involved in metabolizing busulfan. Inflammatory response and activation of coagulation with release of von Willebrand factor, plasminogen activator inhibitor-1 (PAI1), and thrombomodulin were reported to contribute to disease progression [21]. 

Defibrotide is the only approved treatment for severe SOS. Defibrotide stabilizes endothelial cell homeostasis by reducing endothelial-cell activation and damage. It also reduces the plasma levels of plasminogen activator inhibitor-1 (PAI-1), and results in the restoration of the thrombo-fibrinolytic balance [20]. Defibrotide has been successfully used as prophylaxis in patients deemed to be at an increased risk for SOS due to pre-existing liver disease, an underlying condition, or treatment factors [9]. Prophylactic ursodeoxycholic acid has shown efficacy in the reduction in SOS and mortality [1]. 

While the underlying molecular mechanisms are still incompletely understood, genetic variants have been postulated to influence the incidence of SOS for the last two decades [22]. Various pathways have been assessed for their association with SOS. A recent systematic review looked at the influence of glutathione S-transferase genes on pharmacokinetic parameters of busulfan and SOS incidence [23]. To our knowledge, there is no systematic review that summarizes the evidence for all postulated germline genetic predictors for sinusoidal obstruction syndrome. This systematic review describes all identified publications that investigated gene variants associated with SOS in patients of any age who underwent HSCT or were exposed to antineoplastic agents without HSCT. We describe genes and gene variants that were identified and the respective association analyses that were used. 

## 2. Materials and Methods

### 2.1. Study Design 

We performed a systematic literature review following the Preferred Reporting Items for Systematic Review and Meta-Analysis (PRISMA 2009) statement [24] (Supplementary Table S1). We pre-registered the research protocol on PROSPERO (CRD42020215568). 

### 2.2. Study Selection: Eligibility Criteria

We included studies reporting on humans of any age undergoing (allogeneic and autologous) HSCT or treatment with antineoplastic agents without HSCT. We defined antineoplastic agents as all treatments targeting malignant neoplasms including steroids, antihormones, and monoclonal antibodies. We did not use language restrictions. We included studies published from 1 January 1980 to 24 September 2020.

We selected observational studies and longitudinal interventional trials. We initially retained reviews to screen references and then excluded them from the final analysis if no original data were reported. We also excluded opinions, commentaries, conference abstracts, case reports or case series reporting on less than 20 participants, and all reports without original data. We further excluded studies reporting on animal and cell models, and in silico (computer-model) analyses only.

### 2.3. Outcome Definition: Sinusoidal Obstruction Syndrome

We searched for studies with SOS as either the main outcome or outcome with a dedicated association analysis. For patients undergoing HSCT, we included studies with the outcome “sinusoidal obstruction syndrome” as defined by the authors and identified those using established criteria (using the Seattle, ref. [3,8] Baltimore, ref. [4] or new EBMT guidelines [5]). We included patients exposed to antineoplastic agents with the outcome “sinusoidal obstruction syndrome” as defined by the authors. We evaluated the criteria used to diagnose SOS and attributed quality scores (see below: Quality assessment and risk of bias).

### 2.4. Exposures: Genetic Variants

We searched for studies that reported germline genetic variants and their effect on SOS occurrence (i.e., that compared patients with a specific genetic variant to those without). 

### 2.5. Identification of Studies

A systematic literature search was performed using (a) PubMed, (b) EMBASE, (c) Web of Science (Core Collection), (d) Cochrane, (e) CINAHL (EBSCO), and (f) Google Scholar. We searched (g) clinicaltrials.gov for registered studies and searched for published results. We performed a search of the references of identified manuscripts to retrieve further literature. We removed duplicates in the process using the citation manager EndNote (version X8) and Rayyan (https://rayyan.qcri.org; accessed on 1 October 2020) [25]. 

The search strategy was built for all databases using Medical Subject Headings (MeSH) and Title/Abstract (TiAb) terms. We restricted the population of interest to “humans”. For the outcome, we searched for “sinusoidal obstruction syndrome”, and related terms. For the exposure, we used “hematopoietic stem cell transplantation” or “antineoplastic agents” and related terms. For the prognostic factor, we used “genetic variation“, “pharmacogenomic variants”, “pharmacogenetics”, and related terms (see Supplementary Table S2 for the detailed search strategy). We performed the last search update on 24 September 2020. We did not search for unpublished data.

### 2.6. Study Selection

Two authors independently evaluated the eligibility for the inclusion of the identified manuscripts by (i) screening all titles and abstracts, excluding obviously not fitting manuscripts, and then (ii) performing a full-text review of the remaining manuscripts to check for eligibility. For all manuscripts with a discordant assessment of eligibility, the two authors sought agreement through discussion, and where no agreement was reached, a third author judged on eligibility. We used Rayyan for the screening of titles and abstracts. 

### 2.7. Data Extraction

One author extracted data from texts, tables, and graphs. A second author checked the accuracy and completeness of data. Any disagreements were resolved by discussion, and where no agreement was reached, by arbitration of a third author. Unclear or missing data were requested from the corresponding author of the respective manuscripts. We designed a data collection form that included information on the authors; the manuscript; the methodology of germline DNA sequencing and associated quality measures; the study population; the statistical analysis; and results including the strength of association such as relative risks, odds ratios, and hazard ratios. The form was developed and discussed within the review group before full data extraction. 

### 2.8. Quality Assessment and Risk of Bias

The quality assessment was performed by two authors independently using an adapted scoring table based on a previously published scoring system [26] that used the STrengthening the REporting of Genetic Association Studies (STREGA) guidelines [27], an extension of the STROBE Statement [28]. We based the assessment on the reporting, external and internal validity, confounding bias, selection bias, and study power. A study that scored 6 or more out of the 12 points was regarded as high-quality (Supplementary Table S3).

## 3. Results

### 3.1. Study Identification and Selection

**Figure 1:**
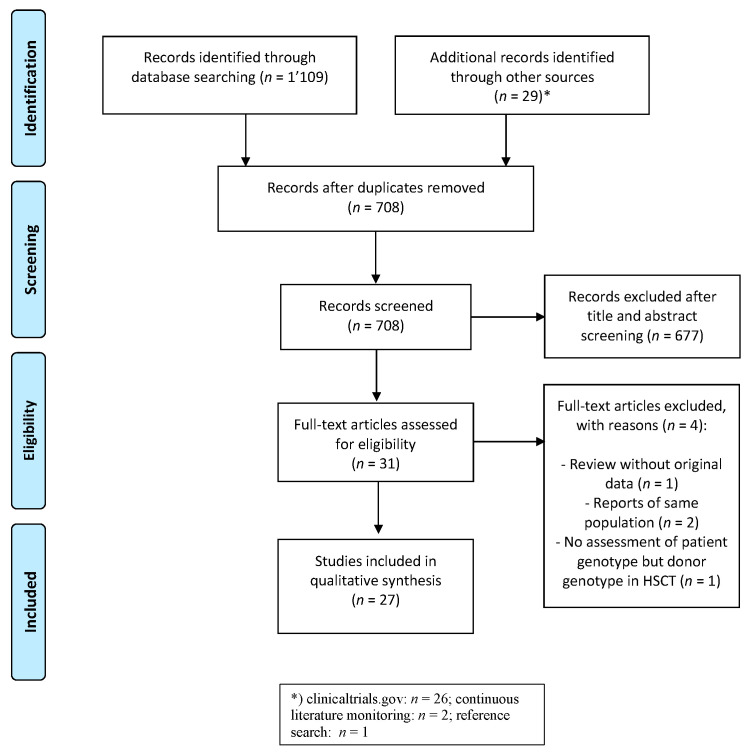
PRISMA Flowchart of the literature review and selection process for genetic predictors for sinusoidal obstruction syndrome after hematopoietic stem cell transplantation and antineoplastic treatment exposure.

We identified 708 unique citations after the removal of duplicates and excluded 677 manuscripts after the title and abstract screening. After assessment of the full text, two reports on subpopulations [29,30] of larger studies were excluded as well as one that assessed HSCT donor genotypes [31] and one review without original data [23]. Finally, we retained 27 manuscripts (Figure 1). 

### 3.2. Characteristics of Included Studies

#### 3.2.1. Study Characteristics

Of the 27 retained manuscripts, 23 were original articles (85%), 2 were short reports (7%) [13,32], and 2 were letters (7%) [22,33]. The study populations (for summary statistics, see Table 1) were collected from retrospective cohort studies (*n* = 23, 85%), two prospective trials (7%) [12,13] and two case-control studies (7%) [22,32]. Eleven manuscripts included patients from Europe (41%), nine from the US or Canada (33%), four from Asia (15%), two from Israel (7%), and one from Turkey (4%). Twenty studies reported data from a single institution (74%), six from multiple sites (22%) and one did not specify clearly (4%). 

#### 3.2.2. Population

Overall, 3150 genotyped patients were included among all the studies. The study population size varied from 18 to 351 with a median of 84. Three studies (11%) included more than 200 genotyped patients [12,13,34]. Eleven studies reported on a mixed population of children, adolescents, and adults (41%); 10 studies reported on children/adolescents (37%); and 6 reported on adult patients (22%). The median age of participants ranged from 4 to 62 years. The proportion of females ranged from 28% to 61% (average 52%). The ethnicity of the study populations was only described in 14 manuscripts (52%), rendering a classification into ethnic groups of the whole population impossible.

#### 3.2.3. Treatment Exposure

Twenty-three studies reported on patients who underwent HSCT (85%). Of those, 19 restricted the exposure to allogeneic HSCT (83%), while four also included autologous HSCT (17%) [22,35,36,37]. Four studies included only HSCT from a sibling donor (17%) [38,39,40,41], the others included various donor types. A wide array of underlying diagnoses were included in most studies on HSCT (*n* = 18, 78%), whereas five studies included only selected diagnoses (two included only acute myeloid leukemia [33,37], two thalassemia [41,42], and one different types of leukemia [40]). Standardized prophylactic treatment was used in four studies (17%) [33,37,43,44], while seven (30%) studies used prophylactic treatment for SOS only in a subgroup: one mentioned ursodeoxycholic acid only (4%) [43]; one heparin only [44]; one defibrotide only [45]; two studies mentioned ursodeoxycholic acid and heparin (9%) [33,37]; one ursodeoxycholic acid and defibrotide [46]; and one study all three prophylactic treatments [47]. It is unclear whether studies not mentioning SOS prophylaxis did not administer prophylaxis or did not describe its use.

Four studies included participants exposed to antineoplastic agents without HSCT (15%). All studies focused on a single underlying disease: two studied acute lymphoblastic leukemia (50%) [12,13], one acute myeloid leukemia (25%) [32], and one colorectal cancer (25%) [48]. No prophylactic treatment was described.

#### 3.2.4. Genotyping

Most studies used a candidate gene approach genotyping based on pre-specified genes (*n* = 26, 96%), only one recent study employed exome-wide sequencing. This is also the only study that attempted to replicate findings in an independent cohort [47]. Six (22%) studies reported on the quality of genotyping by mentioning the number of successful genotyping attempts or cross-validation with a different technique and 11 (41%) took into account Hardy-Weinberg equilibrium when reporting the results.

#### 3.2.5. Outcome

SOS incidence across all cohort-based studies was 16.5% (range 2.3% to 42.9%). The definition of SOS was based on the Seattle or modified Seattle criteria in half of the studies (*n* = 15, 56%), and the Baltimore criteria in five (19%). Various other criteria were used in six studies (22%): two used either clinical criteria or histopathological criteria [22,32], one only histopathological criteria [48], one the National Cancer Institute Common Toxicity Criteria (CTC) versions 2 and 3 [49], and two other clinical criteria based on previously published data [13,44]. One study did not clearly specify the criteria used (4%) [50].

### 3.3. Quality of Studies and Publication Bias

Using an adapted scoring based on the STREGA guidelines, we identified six (22%) studies that we ranked of high quality. The median score of all studies was 5 (range: 2–9 points, Supplementary Table S4). Most studies (*n* = 25, 93%) described clearly their population with exposure and outcome definitions. While all studies reported on the origin of the study population, only two (7%) studies stratified results by study population origin/ethnicity [13,35]. Two studies (7%) performed a power analysis [46] (one of them performed this post hoc [13]). One study replicated the findings in an independent cohort [47]. Clinical characteristics potentially associated with the outcome were described in 15 studies (56%) and 11 (41%) adjusted the genotype-phenotype analysis for clinical variables.

### 3.4. Investigated Genes for Association with SOS after HSCT

#### 3.4.1. Glutathione S-Transferase 

In the 23 studies that included patients with SOS after HSCT (Table 2), variants in 34 different genes were tested, including eight genes identified in the discovery dataset for the WES analysis and tested in the independent replication cohort [47]. The most frequent genes were from the glutathione S-transferase family: *GSTA1* and *GSTM1* (9 studies each), *GSTP1* (7 studies), and *GSTT1* (6 studies). Other members of the glutathione S-transferase family were only investigated in one study each (*GSTO1*, *GSTO2*, and *GSTZ1*). *GSTA1* and *GSTM1* variants were inconsistently associated with SOS: two pediatric studies by Ansari et al. from 2017 and 2020 (replication cohort) reported associations with a slow metabolizer haplotype group of *GSTA1* diplotypes (as defined by the presence of any combination with the *B1b haplotype, *B1a*B1a, or *B2*B1a) [47,51] with odds ratios (OR) of 9.0 (95%-confidence interval [CI] 2.6–31) and 3.1 (CI 1.2–8.0), respectively. The study by Curtis et al. from 2016 performed a gene–gene interaction study with *CTH* but also compared the GSTA1*B*B diplotypes to *A*A/*A*B diplotypes and reported an OR of 10.9 (CI 2.3–51.3) [46]. The *B haplotype, corresponding to the rs3957357 (C > T) or −69 variant, was not found to be associated with SOS in two studies with 84 and 55 adult patients [36,52], and three pediatric and one mainly adult study with 29 to 77 participants [37,41,53,54].

The homozygous deletion of *GSTM1* often referred to as “null genotype” was associated with SOS after allogeneic HSCT with a busulfan-based conditioning regimen in pediatric beta-thalassemia patients (OR 4.3, CI 1.5–12.5, *p* = 0.008) published by Srivastava et al. [42]. This association was not replicated in subsequent studies in predominantly adult [36,37,40,52] and pediatric cohorts [41,51,54], with one pediatric study by Zwaveling et al. showing possible evidence of association (no OR reported, *p* = 0.07) [53]. The *GSTP1* rs1695 (A > G) variant was associated with SOS in a study by Krivoy et al. [37] of 63 adult patients undergoing HSCT for acute myeloid leukemia (no OR reported, *p* = 0.05) but not in other predominantly adult [36,40,52] or pediatric studies [51,53,54]. The *GSTP1* rs1138272(C > T) variant was tested in two studies and the rs614080 (A > G) variant in one study without showing evidence for an association. The *GSTT1* “null genotype” was not associated with SOS in two pediatric [42,53] and four mainly adult [36,37,40,52] studies. *GSTO1*, *GSTO2*, and *GSTZ1* variants were not found to be associated with SOS [36].

#### 3.4.2. Cytochrome P450

Cytochrome P450 family genes were the second group of genes frequently assessed in included studies. *CYP2B6* is an important enzyme in the bioactivation of cyclophosphamide and the *6 haplotype corresponding to rs3745274(G > T) and rs2279343(A > G), was associated with SOS in a study by Rocha et al. (OR 3.5, CI 1.1–10.9) [40]. This study included predominantly adult leukemia patients undergoing HSCT with different regimens, which included cyclophosphamide in 82%. Two other studies including mostly patients treated with cyclophosphamide-containing regimens [36,43] did not find the same association in the CYP2B6*6 haplotype or other assessed variants (*5A haplotype = rs3211371(C > T), rs2279344(A > G), rs2099361(A > C), rs8100458(C > T), rs2014141(A > G)). Further variants assessed by Rocha et al. [40] were not associated with SOS (*2A haplotype = rs8192709(C > T), *4 haplotype = rs2279343(A > G), *5 haplotype = rs3211371(C > T)). Variants in *CYP2C19* [34], which is an important enzyme in cyclophosphamide metabolism, and *CYP2C9* [34,36,43], which has a possible role in busulfan metabolite metabolism, were investigated in a number of studies but no associations were identified. Variants in the ATP-binding cassette subfamily B, member 1 (*ABCB1*), also called multidrug resistance-1 (*MDR1*) gene, were included in two predominantly adult studies without association [37,40]. 

#### 3.4.3. Methylenetetrahydrofolate Reductase 

Variants in the gene coding for the methylenetetrahydrofolate reductase (*MTHFR*) were tested in six studies. All included studies were performed with predominantly adult patients. Goekkurt et al. [52] included 84 patients undergoing busulfan-cyclophosphamide-based allogeneic HSCT for various malignant and non-malignant diseases and identified the rs1801131(CC vs. AC/AA) or 1298A > C variant (OR 9.4, CI 1.1–81.9), but failed to show an association with the rs1801133(CC vs. CT/TT) or 677C > T variant. Efrati et al. [33] performed an analysis on 62 patients undergoing allogeneic HSCT with a busulfan–cyclophosphamide-based conditioning regimen for acute myeloid leukemia. The authors found the rs1801131 (CC vs. AC/AA) variant (no OR published, *p* = 0.0002) and the rs1801133(CC vs.CT/TT) variant (no OR, *p* = 0.0096) associated with SOS. The largest study by Byun et al. [55] included 177 patients undergoing allogeneic HSCT with various conditioning regimens for different diagnoses but was limited by a low proportion of patients with SOS (*n* = 10/177, 5.6%). The authors tested the rs1801133 (CC vs.CT/TT) variant (no OR, *p* = 0.089) and the rs1801133(TT vs.CT/CC) variant (no OR, *p* = 0.234) but did not find an association with SOS. Further studies in 72 to 107 patients did not identify associations of these variants with SOS [38,40,44]. Methotrexate was used as graft-versus-host disease prophylaxis in >90% of patients of the studies that assessed *MTHFR* except in the study by Pihusch et al. [44], which also showed a very low SOS incidence (*n* = 3/89, 3.4%).

#### 3.4.4. Other Liver Enzymes

Kallianpur et al. [35] found an association of the hemostatic iron regulator variant rs1800562(A > G) (*HFE*; RR 3.7; CI 1.2–12.1) and carbamoyl phosphate synthetase I variant rs7422339(CC vs. AC/AA) (*CPS1*, no RR; *p* = 0.04) with SOS in 166 adult patients undergoing autologous or allogeneic HSCT for various malignancies. Sucak et al. [50] did not identify the *HFE* variant rs1799945C > G in 102 adult patients with various underlying diseases. *CPS1* was not included in further studies. The *HPSE* variants rs4364254(TT vs.TC/CC, *p* = 0.004) and rs4693608(AA vs.AG/GG, *p* = 0.038) were associated with SOS in the study by Seifert et al. [45]. Curtis et al. [46] found the cystathionine gamma-lyase (*CTH*) gene variant rs1021737(TT vs.GT/GG) to be associated with SOS in 76 pediatric patients undergoing busulfan-based HSCT for various malignant and non-malignant diseases (OR 10.6, CI 2.2–51.5). Variants in the flavin-containing monooxygenase 3 (*FMO3*) [43] and vitamin D receptor (*VDR*) [40] were not associated with SOS. 

#### 3.4.5. Coagulation and Vascular System

Genes encoding coagulation system proteins were included in four identified studies. Duggan et al. and Pihusch et al. [22,44] did not find an association of prothrombin (*F2*) and factor V (*F5*) variants in mostly adult patients undergoing HSCT with varying conditioning regimens for different underlying diagnoses. The study by Pihusch et al. [44] had a low proportion of participants with SOS (n = 3/89, 3.4%). The study also tested fibrinogen (*FGB*), integrin beta-3 (*ITGB3*), plasminogen activator inhibitor (*SERPINE1*), and the vasculature-associated enzyme angiotensin I-converting enzyme (*ACE*) but did not find an association with SOS. Elbahlawan et al. [49] tested a variant in the cytokine interleukine-1 beta (*IL1B*) gene, which interacts with the endothelium and the coagulation system without association. Lee et al. [39] did not find an association with SOS of the purinergic receptor *P2X* ligand-gated channel 7 gene (*P2RX7*) known to interact with interleukine-1 in 152 mostly adult patients.

#### 3.4.6. Whole Exome Analysis

The only exome-wide association study by Ansari et al. [47] in 87 pediatric patients undergoing busulfan-based allogeneic HSCT for various malignant and non-malignant diseases found eight gene variants associated with SOS in the discovery dataset, of which three were replicated in an independent cohort of 182 pediatric patients *(UGT2B10*, *KIAA1715*, *BHLHE22).* The uridine diphosphate glycosyltransferase 2 family, member 10 *(UGT2B10*, HR 4.7, CI 2.0–11.5) and lunapark *(LNPK* = *KIAA1715*, HR 2.7, CI 1.0–7.5) gene variants were retained in a multivariable model, which controlled for underlying disease, regimen type, and the previously identified risk variants in the *GSTA1* promoter (slow metabolizer haplotypes, HR 3.1, CI 1.2–8.0). 

### 3.5. Investigated Genes for Association with Antineoplastic Agent Exposure

We identified six different genes in four studies investigating SOS after antineoplastic treatments without HSCT (Table 3). Two studies focused on glutathione S-transferase genes: Aplenc et al. [32] assessed variants in *GSTM1*, *GSTP1*, and *GSTT1* in 18 successfully genotyped adult patients receiving ozogamycin-gemtuzumab treatment for relapsed acute myeloid leukemia after HSCT. The study found no association with SOS. Vreuls et al. [48] tested 55 adult patients with metastatic colorectal cancer and oxaliplatin treatment for an association of *GSTM1* and *GSTT1* and found the *GSTM1* “null genotype” to be associated with SOS (no OR published, *p* = 0.03). Lennard et al. examined the TPMT*3A/*3B/*3C haplotypes in 203 patients [12], and Wray et al. examined the same haplotypes and *MTHFR* variants in 351 patients [13] undergoing acute lymphoblastic leukemia treatment. Both studies included patients from prospective trials. The former study found that the prevalence of the TPMT*3A/*3B/*3C alleles was nearly double in the SOS cohort without evidence of association (*p* = 0.11). The other study found no evidence of association of variants in *TPMT* or *MTHFR*. 

## 4. Discussion

This is the first systemtic review that collected the evidence for any postulated germline genetic predictors for sinusoidal obstruction syndrome. We identified 27 studies, 23 on SOS after exposure to HSCT, and 4 after antineoplastic agents without HSCT. Three groups of genes were included in candidate-gene association studies on SOS: genes encoding (i) drug-metabolizing enzymes, mainly glutathione S-transferases (GST), cytochrome P450 family enzymes (CYP), and *MTHFR;* (ii) other enzymes mainly active in the liver; and (iii) coagulation factors and other proteins closely interacting with the coagulation or vascular system. Variants in nine different genes showed an association with SOS in the included candidate-gene association studies *(CPS1*, *CTH*, *CYP2B6*, *GSTA1*, *GSTM1*, *GSTP1*, *HFE*, *HPSE*, *MTHFR).* Of those, only two were associated in more than one study: *GSTA1* in three studies [46,47,51] and *MTHFR* in two studies [33,52]. Additionally, the study using WES data [47] identified variants in eight genes, of which two were retained after a stepwise selection using a multivariate Cox regression model after replication in an independent cohort (*UGT2B10* and *KIAA1715* = *LNPK*). 

*GSTA1* variants were frequently included in genotype–phenotype association analyses due to their importance in drug metabolism, particularly in electrophilic chemotherapies such as busulfan. An association of these chemotherapies with SOS has been known for more than three decades [56]. The metabolization of busulfan is performed in the liver through conjugation with glutathione both spontaneously and by catalysis particularly of the alpha1 isoform (*GSTA1*), followed by mu1 (*GSTM1*) and pi1 (*GSTP1*) [57]. Three of the nine studies identified an association of *GSTA1* variants with SOS, while six did not. The first reason for this discrepancy might be the differences in the genetic variants that were compared: different genetic variants in promoter regions have been shown to modify the expression of the metabolic enzyme to varying degrees [58]. Slow metabolizer haplotypes were only tested by Ansari et al. [47,51]. The slow metabolizer haplotypes were associated with SOS in multivariable association analyses taking into account underlying disease and type of conditioning regimen. Curtis et al. [46] found an association of the *B*B haplotype with SOS. Other studies compared the *B haplotypes but did not find an association. Second, a limited number of participants included in many studies might have impacted the ability to identify associations and the precision of effect sizes with large confidence intervals. The studies reporting no association with pediatric participants included only 29 to 77 participants. Third, *GSTA1* haplotypes have been consistently associated with busulfan pharmacokinetics [51,59]. However, *GSTA1* was reported to be more important for busulfan clearance in young versus older children due to the maturation of other pathways for busulfan clearance, with older age rendering *GSTA1* less important [60,61]. Infants also had a more variable clearance than older patients [62]. A limitation of all these studies is that busulfan clearance was not included in the models testing *GSTA1* variants with SOS. Therefore, it remains unclear if *GSTA1* has an association with SOS beyond its effect on busulfan clearance. 

Variants in two other genes from the glutathione S-transferase family were associated with SOS in one study each, while other studies failed to report an association. *GSTM1* “null genotype” was associated with SOS in one [42] study, while seven did not reveal an association [36,37,40,41,51,52,54]. *GSTP1* rs1695(A > G) was found associated with SOS in one study [37], while six studies showed no association [36,40,51,52,53,54]. Possibly, the heterogeneity between these studies in terms of age at HSCT, HSCT conditioning regimen, and underlying disease might have contributed to the varying results. A recent systematic review with a meta-analysis of glutathione S-transferase genes was performed by Kim et al. [23]. The authors included nine studies on GSTA1*A*A versus *B haplotypes, seven studies on *GSTM1* “null genotype”, and five studies on *GSTP1*. They showed an association of the GSTA1*B haplotype with the area under the curve of intravenous busulfan but failed to show an association of glutathione S-transferases with SOS. However, some of the studies we identified were not included in the analysis by Kim et al. [23]. The studies by Ansari et al. 2013, 2017 and 2020 [30,47,51] were not assessed, while some studies that they included did not meet our inclusion criteria as they reported low patient numbers (<20 participants) or no patients with the outcome of interest. While *GSTM1* “null genotype” is often cited as a risk factor for SOS [7,63], our review showed that this association is inconsistently reported and needs further evaluation. 

*CYP2B6* variants were inconsistently associated with SOS. *CYP2B6* is involved in the metabolization of the antineoplastic drug cyclophosphamide, which is often used uring the conditioning regimen [64]. *CYP2B6* has a variable expression between individuals due to genetic and treatment-related factors (e.g., induction by cyclophosphamide or inhibition by thiotepa). The cytochrome P450 gene CYP2B6*6 haplotype was associated with SOS in the study by Rocha et al. [40]. This was also the largest study including *CYP2B6* with 107 mainly adult patients. Two other studies with 66 pediatric and 55 adult patients did not show an association of this haplotype with SOS [36,43]. These two studies included patients treated with busulfan and cyclophosphamide, while the study by Rocha et al. [40] included conditioning regimens mostly containing cyclophosphamide with other agents. The role of *CYP2B6* variants in SOS remains unclear.

*MTHFR* variants were associated with SOS in two studies [33,52], but not in four [38,40,44,55]. *MTHFR* is coding for a key enzyme involved in the homocysteine and folate metabolism [65]. Elevated levels of homocysteine were shown to be associated with vascular injury and thrombosis [66], which provided a rationale for including *MTHFR* genetic variants in studies on SOS. Methotrexate is a folic acid antagonist and used as graft-versus-host disease prophylaxis in many HSCT conditioning regimens [67]. The importance of *MTHFR* in the folate metabolism was another reason that this gene was investigated. We found two possible explanations for the differences in associations: First, the incidence of SOS varied widely between the studies. SOS was seen in 15% [33] to 42.9% [52] of participants in studies with, and 3.4% [44] to 14% [40] in those without association, illustrating heterogeneity in the baseline risk for SOS in the different populations. Second, we found that studies showing an association included patients undergoing busulfan-based regimens, while studies showing no association included various conditioning regimens. In conclusion, *MTHFR* variants might play a role in high-risk patients and after busulfan-based conditioning. 

Several other liver enzymes were analyzed in included studies. The *HFE* gene rs1800562(A > G) variant was associated with hemochromatosis previously, which leads to excessive iron accumulation in the liver and hepatocyte injury. That variant was associated with SOS in the study by Kallianpur et al. [35]). Sucak et al. [50] tested another hemochromatosis-associated variant rs1799945(C > G) without association with SOS. Variants in three other genes were associated with SOS in one study without testing in further studies: *CTH* was associated with SOS in the study by Curtis et al. [46] but a wide confidence interval indicated low precision of the estimate. *CTH* is involved in glutathione synthesis. Glutathione is depleted by busulfan and cyclophosphamide. *CPS1* codes for the enzyme necessary for the first step of the urea cycle and metabolization of excess nitrogen. It was hypothesized that the s7422339(CC vs. AC/AA) variant in *CPS1* might lead to reduced antioxidant efficiency. Kallianpur et al. found an association of that variant with SOS, which was not investigated in further studies [35]. The protein coded by *HPSE* cleaves heparan sulfate proteoglycans, which are part of the extracellular matrix and are involved in inflammation, angiogenesis, and tissue repair. Two *HPSE* variants were associated with SOS in the study by Seifert et al. [45]. The importance of these genetic variants remains unclear without further replication. Genes coding for proteins relevant to the coagulation or vascular system were investigated in four studies, none of them found an association [22,39,44,49]. 

The study by Ansari et al. 2020 [47] showed in their exome-wide analysis an association of *UGT2B10* with SOS in the discovery dataset and replication in independent patients. The association remained when assessing the gene variant in different subgroups of one and multiple alkylating agents. *UGT2B10* is involved in detoxifying various compounds through glucuronidation [68] and is exclusively expressed in liver tissue [69]. The other gene identified and replicated in the independent cohort was *KIAA1715* = *LNPK*. This gene encodes the endoplasmic reticulum (ER) junction formation factor involved in the structural organization of the endoplasmic reticulum and associated with neurodegenerative disease. *LNPK* was only associated with SOS in patients receiving two or more alkylating agents. 

In the studies including antineoplastic agents without HSCT as exposure, two large studies by Lennard et al. [12] and Wray et al. [13] did not find an association of *TPMT* variants with SOS after acute lymphoblastic leukemia (ALL) treatment including patients exposed to thioguanine. The latter study did also test *MTHFR* variants without evidence of association. The study by Aplenc et al. [32] on 18 genotyped patients with ozogamicin-gemtuzumab treatment did find an OR of 4 of patients with the GSTP1*B haplotype but did not give further details on the strength of the association due to the low patient numbers. The study by Vreuls et al. [48] on 55 adult patients with metastatic colorectal cancer treated with oxaliplatin found an association with the *GSTM1* “null genotype”. Only histopathological criteria were used to identify SOS, which makes this study difficult to compare to others in our review. 

The quality of studies included in this review was overall low, which is a limitation of the presented data in this review. There were very few studies that stratified or adjusted the analysis for ethnicity (n = 2, 7%), performed a power calculation for the sample size (n = 2, 7%), corrected results for multiple testing (n = 1, 4%), or replicated results in a separate sample (n = 1, 4%). We then found large heterogeneity between studies. Patients varied in terms of underlying diagnoses, types of conditioning regimens or antineoplastic agents without HSCT, and outcome definitions. The age range, definition, and prevalence of outcomes varied between studies. Finally, prophylactic treatments were used in some studies in a standardized way, while most included patients with several prophylactic treatments. The heterogeneity of patients within studies was also large. Many studies included pediatric and adult patients, different underlying diagnoses, different conditioning regimens, and different prophylactic treatments in the same study sample. We estimated that the heterogeneity within and between studies is too large to perform a meta-analysis. Additionally, many gene variants were only assessed in one or only a few studies.

The strengths of our review are the broad scope and the number of studies that we identified and summarized. We applied a stringent pre-published protocol, including a data collection form, quality and bias assessment with a pre-defined threshold for high versus low-quality studies. We did not restrict our literature search for language and screened a large number of databases. We used two assessors for the screening and quality evaluation process and a third assessor for arbitration. Data collection was checked by a second author. 

## 5. Conclusions

The strongest evidence for an association of genotypes with SOS was found for *GSTA1* variants (slow metabolizer haplotypes). Still, it is unclear whether *GSTA1* affects SOS beyond its influence on busulfan clearance. Some evidence was found for *MTHFR* variants in high-risk patients after busulfan-based conditioning regimens. Most included studies used a candidate-gene approach. Only one study used an exome-wide approach, which was also the only study with replication of results in an independent patient cohort. A wide number of genes was either inconsistently associated with SOS or only studied in one cohort. 

Future studies should include sufficiently large samples of patients with ideally a single underlying disease using one treatment protocol. Power analyses are essential to design appropriate studies. An ideal setup are clinical trials with ancillary genetic studies using a clearly defined patient population. It is also important to adequately assess and adjust for relevant clinical covariates and ethnicity. To be able to compare future studies, standardized outcome measures should be employed. Finally, future studies should include replication populations that are similar to the discovery dataset to assess the external validity of identified associations. 

## Figures and Tables

**Table 1:** Summary characteristics of studies reporting on genetic predictors of SOS (*n* = 27).

Characteristics	*n*	Proportion (%)
Centers included		
monocentric	20	74.1
multicentric	6	22.2
unclear	1	3.7
Location		
Europe	11	40.7
North America	9	33.3
Asia	4	14.8
Others	3	11.1
Study design		
cohort	23	85.2
prospective trial	2	7.4
case-control	2	7.4
Sample size		
median, IQR (n)	84	65–142
0–50	3	11.1
51–100	13	48.1
101–150	4	14.8
151–200	4	14.8
201 and more	3	11.1
Age group at treatment		
children and adolescents only	10	37
children, adolescents, and adults	11	40.7
adults only	6	22.2
Treatment exposure		
allogeneic HSCT, busulfan-based	10	37
allogeneic HSCT, various regimens	9	33.3
autologous and allogeneic HSCT	4	14.8
non-HSCT	4	14.8
Outcome		
incidence cohort-based samples (mean %, range %)	16.5	2.3–42.9
(modified) Seattle criteria	15	55.6
Baltimore criteria	5	18.5
other criteria/unspecified	7	25.9
Association analysis		
candidate gene analysis	26	96.3
genome/exome wide analysis	1	3.7

Legend: HSCT, hematopoietic stem cell transplantation; IQR, interquartile range; *n*, number.

**Table 2:** Summary of 23 studies on genetic variants and their association with sinusoidal obstruction syndrome after allogeneic hematopoietic stem cell transplantation. Publications are listed in chronological order of publication.

Lead Author, Journal Year	Study Design	Location	Population (Diagnoses, Age)	Exposure, Location	*n* (SOS/Total)	Genes/Region	Variants Investigated	OR/ RR (CI)	*p*-Value
Duggan C, et al. Bone Marrow Transplant. 1999. [22]	Candidate-gene; case-control	St James’s Hospital and Trinity College Dublin, Ireland	Unclear diagnoses, median age 29 years (range 4–55)	AlloHSCT and autoHSCT with various regimens (Bu, Cy, Mel, TBI, others)	22/287 (7.7%), genotyped: 15/51 (29.4%)	*F2*	rs1799963(GA vs. GG)	-	*p* = 0.05
						*F5*	rs6025(GG vs. AG/AA)	-	*p* = 0.05
Pihusch M, et al. Transplantation. 2004 [44]	Candidate-gene; cohort	José-Carreras transplantation unit Munich, Germany	Various malignant and non-malignant diagnoses; median age 43 years (range 14–62)	AlloHSCT with various regimens (Bu, Cy, Mel, TBI, others)	3/89 (3.4%)	*F2*	rs1799963(G > A)	“no effect”	-
						*F5*	rs6025(G > A)	“no effect”	-
						*MTHFR*	rs1801133(C > T)	“no effect”	-
						*ITGB3*	rs591(C > T)	“no effect”	-
						*FGB*	rs1800790(G > A)	“no effect”	-
						*SERPINE1*	rs1799889(4G allele)	(83.3% vs. 55.1%)	NS
						*ACE*	rs1799752(D allele)	“no effect”	-
Srivastava A, et al. Blood. 2004 [42]	Candidate-gene; cohort	Hôpital Robert Debré, Paris, France	Beta-thalassemia major; median age 6 years (range 2–16)	Busulfan–cyclophosphamide-based alloHSCT	33/114 (28.9%)	***GSTM1***	**“null genotype” ‡**	**OR 4.3 (1.5–12.5) †**	***p* = 0.008 †**
						*GSTT1*	“null genotype”‡	OR 0.6 (0.2–1.9) †	p = 0.4 †
Kallianpur AR et al. Bone Marrow Transplant. 2005 [35]	Candidate-gene; cohort	Multicentric, two centers in Nashville, Tennessee, USA	Various hematological and solid neoplasms; mean age 44 years (range 19–64)	AlloHSCT and autoHSCT with various regimens (Bu, Cy, TBI, others)	30/166 (18.1%)	***HFE***	**rs1800562(A > G)**	**RR 3.7 (1.2–12.1); RR 1.7 (0.4–6.8) for heterozygotes; RR 8.6 (1.5–48.5) for homozygotes †**	***p* = 0.01 †**
						***CPS1***	**rs7422339(CC vs. AC/AA)**	**-**	***p* = 0.038**
Elmaagacli AH, et al. Bone Marrow Transplant. 2007 [34]	Candidate-gene; cohort	University Hospital of Essen, Germany	Various hematological neoplasms incl. lymphomas; median age 41 years (range 17–67)	AlloHSCT with various regimens (Bu, Cy, TBI, others)	20/286 (7%)	*CYP2C19*	Poor vs. intermediate/extensive metabolizers (rs4244285(AA vs. AG/GG) rs4986893(AA vs. AG/GG))	-	NS
Goekkurt E, et al. Anticancer Res. 2007 [52]	Candidate-gene; cohort	University Hospital Hamburg, Germany	Various hematological malignancies and non-malignant diagnoses; median age 39.5 years (range 16–59)	Busulfan–cyclophosphamide-based alloHSCT	36/84 (42.9%)	*GSTA1*	* B vs. * A haplotypes	-	NS
						*GSTM1*	“null genotype”‡	-	NS
						*GSTP1*	rs1695(A > G)	-	NS
						*GSTT1*	“null genotype”‡	-	NS
						***MTHFR***	rs1801133(C > T)	-	NS
							**rs1801131(A > C)**	**OR 9.4 (1.1–81.9) †**	***p* = 0.048 †**
Kim I, et al. Annals of Hematol. 2007 [38]	Candidate-gene; cohort	Seoul National University College of Medicine, South Korea	Hematological malignancies and aplastic anemia; median age 36 year (range 16–52)	AlloHSCT with various regimens (Bu, Cy, TBI)	11/72 (15.3%)	*MTHFR*	rs1801133(C > T)	-	*p* = 0.4
							rs1801131(A > C)	-	*p* = 0.48
Lee KH, et al. Haematologica. 2007 [39]	Candidate-gene; cohort	Seoul National University Hospital, South Korea	Hematological malignancies incl. lymphomas and aplastic anemia; median age 40 years (range 16–70)	AlloHSCT with various regimens (Bu, Cy, Mel, TBI, others) from HLA-matched sibling donors	19/152 (12.5%)	*P2RX7*	rs3751143(A > C)	-	*p* = 0.78
Zwaveling J, et al. Therapeut Drug Monitor. 2008 [53]	Candidate-gene; cohort	Multicentric, pediatric Leiden and Utrecht University Medical Centers, Netherlands	Hematological malignancies and non-malignant diagnoses; median age 5 years (range 0.2–23)	Busulfan-based alloHSCT with various other agents (Cy, Mel, others)	15/77 (19.5%)	*GSTA1*	rs3957357(C > T)	-	-
						*GSTM1*	“null genotype” ‡	-	*p* = 0.07
						*GSTP1*	rs1695(A > G)	-	-
						*GSTT1*	“null genotype” ‡	-	-
Johnson L, et al. J Clin Pharmacol. 2008 [54]	Candidate-gene; cohort	University of Minnesota, USA	Malignant and nonmalignant diagnoses; median age 5.6 years (range 0.1–18.3)	Busulfan-based alloHSCT with various other agents (Cy, others)	3/29 (10.3%)	*GSTA1*	* B vs. * A haplotypes	-	NS
						*GSTM1*	“null genotype” ‡	-	NS
						*GSTP1*	rs1695(A > G)	-	NS
							rs1138272(C > T)	-	NS
Rocha V, et al. Leukemia. 2009 [40]	Candidate-gene; cohort	Hôpital Saint Louis, Paris, France	Acute and chronic leukemia; median age 35 years (range 3–56)	AlloHSCT with various regimens (Bu, Cy, Mel, TBI, others) from HLA-matched sibling donors	15/107 (14%)	***CYP2B6***	* 2A haplotype	-	NA
							* 4 haplotype	-	NA
								* 5 haplotype	-	NA
								* 6 haplotype	**OR 3.49 (1.12–10.88) †**	***p* = 0.03 †**
						*GSTM1*	“null genotype” ‡	-	NA
						*GSTP1*	rs1695(AA vs. AG/GG)		NA
						*GSTT1*	“null genotype” ‡	-	NA
						*ABCB1*	rs1045642(CC vs. CT/TT)	-	NA
						*MTHFR*	rs1801133(CC vs. CT/TT)		NA
						*VDR*	Apal (rs7975232)	-	NA
							BsmI (rs1544410)		NA
							TaqI (rs731236)	-	NA
Elbahlawan L, et al. J Ped Hem Oncol. 2012 [49]	Candidate-gene; cohort	St Jude Children’s Research Hospital, USA	Malignant and non-malignant diagnoses; median age 10.1 years (range 1–19.6)	AlloHSCT with various regimens (Bu, Cy, TBI, others) from HLA-matched donors	5/76 (6.6%)	*IL1B*	rs16944(A > G)	-	*p* = 0.18
Sucak GT, et al. Ann Hematology. 2012 [50]	Candidate-gene; cohort	Gazi University, Ankara, Turkey	Malignant and non-malignant diagnoses; median age 27.5 years (range 16–64)	AlloHSCT with various regimens (Bu, Mel, TBI, others)	22/102 (21.6%)	*HFE*	rs1799945(C > G)	-	*p* > 0.05
Krivoy N, et al. Curr Drug Safety. 2012 [37]	Candidate-gene; cohort	Technion-Israel Institute of Technology; Haifa, Israel	Acute myeloid leukemia; median age 39.2 years (SD 12.3)	Busulfan–cyclophosphamide-based autoHSCT and alloHSCT	8/63 (12.7%)	*ABCB1*	rs1045642(C > T)	-	NS
							rs2032582(G > T/A)	-	NS
						*GSTA1*	rs3957357(C > T)	-	NS
						*GSTM1*	“null genotype” ‡	-	NS
						***GSTP1***	**rs1695(A > G)**	**-**	***p* = 0.05**
						*GSTT1*	“null genotype” ‡	-	NS
Uppugunduri CRS, et al. Pharmacogenom J. 2014 [43]	Candidate-gene; cohort	CHU Sainte-Justine, Montreal, Canada	Malignant and non-malignant diagnoses; median age 6.9 years (range 0.1–19.9)	Busulfan–based alloHSCT with various other agents (Cy, Mel, TBI, others)	8/66 (12.1)	*CYP2B6*	rs3211371(C > T)	-	NS
							rs3745274(G > T)	-	NS
						*CYP2C19*	rs4244285(G > A)	-	NS
							rs12248560(C > T)	-	NS
						*CYP2C9*	rs1799853(C > T)	-	NS
							rs1057910(G > A)	-	NS
						*FMO3*	rs2266780(A > G)	-	NS
							rs2266782(G > A)	-	NS
							rs1736557(A > G)	-	NS
Efrati E, et al. Bone Marrow Transplant. 2014 [33]	Candidate-gene; cohort	Technion-Israel Institute of Technology; Haifa, Israel	Acute myeloid leukemia; adult cohort	Busulfan–cyclophosphamide-based alloHSCT (with TBI in one)	9/62 (15%)	***MTHFR***	**rs1801133(CC vs. CT/TT)**	**-**	***p* = 0.0096**
							**rs1801131(CC vs. AC/AA)**	-	***p* = 0.0002**
Seifert C, et al. J. Cancer Res. Clin. Oncol. 2015 [45]	Candidate-gene; cohort	Jena University Hospital, Germany	Malignant and non-malignant diagnoses; median age 14 years, (range 0–29)	AlloHSCT with various regimens (Bu, Cy, Mel, TBI)	12/160 (7.5%)	***HPSE***	**rs4693608(AA vs. AG/GG)**	**-**	***p* = 0.038**
							**rs4364254(TT vs. TC/CC)**	**-**	***p* = 0.004**
							**rs4693608(AA)and rs4364254(TT) †**	**4.06 (1.14–14.4) †**	***p* = 0.03 †**
Ansari M, et al. Bone Marrow Transplant. 2016 [41]	Candidate-gene; cohort	San Raffaele Institute, Milan, Italy	Thalassemia intermedia (20.5%) and thalassemia major (79.5%); median age 8 years (range 1.5–17)	Busulfan–cyclophosphamide-based alloHSCT from HLA-matched sibling donors	1/44 (2.3%)	*GSTA1*	* B vs. * A haplotypes using rs3957357(C > T)	-	NS
						*GSTM 1*	“null genotype” ‡	-	NS
Byun JM, et al. PloS One. 2016 [55]	Candidate-gene; cohort	Seoul National University Hospital, South Korea	Hematological malignancies incl. lymphomas and aplastic anemia; median age 37.8 years (SD 12.5)	AlloHSCT with various regimens (not further specified)	10/177 (5.6%)	*MTHFR*	rs1801133(TT vs. CT/CC)	-	*p* = 0.234
Huezo-Diaz Curtis *p*, et al. Pharmacogenomics J. 2016 [46]	Candidate-gene; cohort	CHU Sainte-Justine, Montreal, Canada	Malignant and non-malignant diagnoses; median age 6.4 years (range 0.1–19.9)	Busulfan-based alloHSCT with various other agents (Cy, Mel, others)	9/76 (11.8%)	***CTH***	**rs1021737(TT vs. GT/GG)**	**OR 10.6 (2.2–51.5)**	***p* = 0.003**
							rs648743(C > T)	-	NS
						***GSTA1***	* B* B vs. * A* B/* A* A haplotypes	**OR 10.9 (2.3–51.3)**	***p* = 0.007**
Ansari M, et al. Oncotarget. 2017; [51] → includes all patients from: [29] and [30]	Candidate-gene; cohort	Multicentric: Geneva, Leiden, Montreal, Paris, Toronto	Malignant and non-malignant diagnoses; median age 5.8 years (range 0.1–19.9)	Busulfan-based alloHSCT with various other agents (Cy, Mel, others)	14/138 (10%)	***GSTA1***	**Slow metabolizer haplotypes (group IV)**	**OR 9.0 (2.6–31) †**	***p* = 0.001 †**
						*GSTM1*	“null genotype” ‡	-	NA
						*GSTP1*	rs1695(A > G)	-	NA
							rs1138272(C > T)	-	NA
Ansari M, et al. Biology of Blood and Marrow Transplantation. 2020 [47]	Exome-wide association analysis with replication in an independent sample; cohort	Discovery cohort: CHU Sainte-Justine, Montreal, Canada; replication cohort: multicentric	Malignant and non-malignant diagnoses; median age discovery: 7.4 years (range 0–23.5); replication: 4.7 years (range 0–21)	Busulfan-based alloHSCT with various other agents (Cy, Mel, others)	Discovery: 12/87 (13.8%); replication: 27/182 (14.8%)	***UGT2B10***	**rs17146905A > G**	**OR 8.4 (3.0–23.9)**	***p* = 7 × 10^−6^ (replication *p* = 0.0004 †)**
						***KIAA1715* = *LNPK***	**rs2289971T > C**	**OR 10.2 (3.3–31.9)**	***p* = 3 × 10^−6^ (replication *p* = 0.05 †)**
						*BHLHE22*	rs16931326G > A	OR 8.9 (2.9–26.9)	*p* = 1.1 × 10^−5^ (replication *p* > 0.05 §)
						*HADH*	rs17511319A > G	OR 30.5 (5.9–158.6)	*p* = 1.2 × 10^−5^ (replication *p* = 0.05)
						*ZNF608*	rs75323508 C > T	OR 9.9 (3.0–32.8)	*p* = 1.3 × 10^−5^ (replication *p* = 0.4)
						*AMPH*	rs2810T > C	OR 8.9 (2.9–26.9)	*p* = 1.1 × 10^−5^ (replication *p* = 0.9)
						*FAT3*	rs11823754G > T	OR 10.7 (3.6–31.7)	*p* = 8.3 × 10^−7^ (replication *p* = 1.0)
						*AGPAT3*	rs11537798A > G	OR 9.9 (3.0–32.8	*p* = 1.3 × 10^−5^ (replication *p* = 0.1)
						***GSTA1***	**Slow metabolizer haplotypes (group IV)**	**OR 3.1 (1.2–8.0) in replication cohort †**	**replication cohort only: *p* = 0.02 †**
Terakura S, et al. Int J Hematol. 2020 [36]	Candidate-gene; cohort	Nagoya University Hospital, Japan	Hematological malignancies incl. lymphomas; median age 38 years (21–67)	Busulfan–cyclophosphamide based autoHSCT and alloHSCT	8/55 (14.5%)	*CYP2B6*	rs3745274(G > T)	-	NS
							rs2279344(A > G)	-	NS
							rs2099361(A > C)	-	NS
							rs8100458(C > T)	-	NS
							rs2014141(A > G)	-	NS
						*CYP2C9*	rs1799853	-	NS
							rs1057910(A > C)	-	NS
						*CYP2C19*	rs4986893 (G > A)	-	NS
							rs4244285(G > A)	-	NS
						*GSTA1*	* B vs. * A haplotype (rs4715326)	-	NS
						*GSTM1*	“null genotype” ‡	-	NS
						*GSTO1*	rs4925(A > C)	-	NS
							rs11191972(C > T)	-	NS
						*GSTO2*	rs156697(A > G)	-	NS
							rs2297235(A > G)	-	NS
						*GSTP1*	rs1695(A > G)	-	NS
							rs614080((A > G)	-	NS
						*GSTT1*	“null genotype” ‡	-	NS
						*GSTZ1*	rs2270423(A > G)	-	NS

**Legend:** bold font, significant association; †, after adjustment for clinical covariables (multivariable regression analysis); §, not retained in multivariable Cox regression model; ‡, “null genotype” is used for genotypes with absence of enzyme activity; ALL, acute lymphoblastic leukemia; allo, allogeneic; AML, acute myeloid leukemia; auto, autologous; BM, bone marrow; Bu, Busulfan; CI, 95%-confidence interval; Cy, cyclophosphamide; HSCT, hematopoietic stem cell transplantation; HLA, histocompatibility lymphocyte antigen; Mel, melphalan; n, number; OR, odds ratio; NA, not available; NS, not significant; RR, relative risk; SOS, sinusoidal obstruction syndrome; TBI, total body irradiation. Gene names: *ABCB1* = *MDR*, multidrug-resistance gene; *ACE*, angiotensin I converting enzyme; *AGPAT3*, 1-acyl-glycerol 3-phosphate o-acyltransferase 3; *AMPH*, amphiphysin; *BHLHE22*, basic helix-loop-helix family, member 22; *CPS1*, carbamoyl phosphate synthetase I; *CTH*, cystathionine gamma-lyase; *CYP2B6*, cytochrome P450 B6; *CYP2C19*, cytochrome P450 C19; *CYP2C9*, cytochrome P450 C9; *F2*, coagulation factor 2 = prothrombin; *F5*, coagulation factor 5; *FAT3*, Fat atypical cadherin 3; *FGB*, Fibrinogen B beta polypeptide; *FMO3*, flavin-containing monooxygenase 3; GST, glutathione S transferase; *GSTA1*, glutathione S transferase A1; *GSTM1*, glutathione S transferase M1; *GSTO1*, glutathione S transferase O1; *GSTO2*, glutathione S transferase O2; *GSTP1*, glutathione S transferase P1; *GSTT1*, glutathione S transferase T1; *GSTZ1*, glutathione S transferase Z1; *HADH*, *3-hydroxyacyl-CoA dehydrogenase; HFE*, homeostatic iron regulator; *HPSE*, heparanase; *IL1B*, interleukin 1-beta; *ITGB3*, integrin beta-3; *KIAA1715* = *LNPK*, lunapark; *MTHFR*, methylenetetrahydrofolate reductase; *P2RX7*, purinergic receptor P2X, ligand-gated ion channel, *7; SERPINE1*, plasminogen activator inhibitor-1; *TPMT*, thiopurine S-methyltransferase; *UGT2B10*, uridine diphosphate glycosyltransferase family 2 member B10; *VDR*, vitamin D receptor; *ZNF608*, Zinc Finger Protein 608.

**Table 3:** Summary of 4 studies on genetic variants and their association with sinusoidal obstruction syndrome after antineoplastic agents without hematopoietic stem cell transplantation. Publications are listed in chronological order of publication.

Lead Author, Journal Year	Study Design	Location	Population (Diagnoses, Age)	Exposure, Location	*n* (SOS/Total)	Genes/Region	Variants Investigated	OR (CI)	*p*-Value
Aplenc R, et al. Acta Haematologica. 2003 [32]	Candidate-gene; case-control	University of Washington Medical Center, Seattle, USA	Relapsed AML; mean age 45.4 years	Gemtuzumab for relapsed disease after HSCT (SOS not primarily associated with HSCT)	11/21 (52%) Genotyped: 9/18 (50%)	*GSTM1*	“null genotype” ‡	-	NS
						*GSTT1*	“null genotype” ‡	-	NS
						*GSTP1*	*B haplotype	OR 4 (NA)	NS
							*C haplotype	-	NS
						*NQ01*	*2 haplotype	-	NS
Lennard L, et al. Clin. Pharmacol. Ther. 2006 [12]	Candidate-gene; case-control based on prospective trial	Multicentric, USA	Acute lymphoblastic leukemia; median age 4 years (range 1–16)	Treatment according to protocols CCG-ALL97 (*n* = 33/393 with SOS, 8%) and CCG-ALL99 (*n* = 49/355 with SOS, 14%)	50/203 (24.6%)	*TPMT*	*3A/*3C haplotypes	-	*p* = 0.11
Vreuls CPH, et al. Brit J Cancer. 2013 [48]	Candidate-gene; cohort	Maastricht University Medical Centre, NL	Patients with metastatic colorectal cancer; mean age 62 years (range 40–81)	Initial partial hepatic resection and treatment with oxaliplatin	32/55 (58%)	***GSTM1***	**“null genotype” ‡**	**-**	***p* = 0.026 †**
						*GSTT1*	“null genotype” ‡	-	NS
Wray L, et al. Pediatr Blood Cancer. 2014 [13]	Candidate-gene; prospective trial	Children’s Hospital of Philadelphia, USA	Acute lymphoblastic leukemia; pediatric patients (range 1–10 years)	Treatment according to protocol CCG-1952	79/351 (22.5%)	*TPMT*	*3A haplotype	OR 0.7 (0.3–1.6) †	NS †
							*3B haplotype	OR 1.0 (0.4–2.6) †	NS †
							*3C haplotype	OR 0.7 (0.2–1.8) †	NS †
						*MTHFR*	rs1801133(CC vs.CT/TT)	OR 0.9 (0.3–2.4) †	NS †
							rs1801131(CC vs.AC/AA)	OR 1.4(0.5–3.8) †	NS †

**Legend:** bold font, significant association; †, after adjustment for clinical covariables (multivariable regression analysis); ‡, “null genotype” is used for genotypes with absence of enzyme activity; ALL, acute lymphoblastic leukemia; allo, allogeneic; AML, acute myeloid leukemia; allo, allogeneic; BM, bone marrow; Bu, Busulfan; CI, 95%-confidence interval; HSCT, hematopoietic stem cell transplantation; HLA, histocompatibility lymphocyte antigen; NA, not available; NS, not significant; OR, odds ratio; SOS, sinusoidal obstruction syndrome; TBI, total body irradiation. Gene names: GSTA1, glutathione S transferase A1; *GSTM1*, glutathione S transferase M1; *GSTP1*, glutathione S transferase P1; *GSTT1*, glutathione S transferase T1; *MTHFR*, methylenetetrahydrofolate reductase; *NQ01*, NAD(P)H Quinone Dehydrogenase 1; *TPMT*, thiopurine S-methyltransferase.

## Data Availability

Data sharing is not applicable to this article.

## Supplementary Materials

The following are available online at https://www.mdpi.com/article/10.3390/jpm11050347/s1. Table S1. PRISMA checklist for the systematic review of genetic predictors for sinusoidal obstruction syndrome. Table S2. Detailed search strategy to identify manuscripts of genetic risk analysis with sinusoidal obstruction syndrome (PubMed). Table S3. Quality assessment tool: Detailed description for scoring (0–12 possible points; based on STREGA checklist1 and adapted from Zazuli et al. and Leusink et al.). Table S4. Quality assessment of 27 included studies on genetic predictors for sinusoidal obstruction syndrome after HSCT or chemotherapy; after agreement was reached between authors.
