# Supplementary material for: Genetic Predictors for Sinusoidal Obstruction Syndrome—A Systematic Review
Source: J Pers Med. 2021 Apr 26;11(5):347. doi: 10.3390/jpm11050347 (PMC8145271; doi:10.3390/jpm11050347)
Supplement: Supplementary file 1 [file jpm-11-00347-s001.zip › A03_Waespe_SOS PGX Review_Supplement_FINAL.pdf]

Genetic predictors for sinusoidal obstruction syndrome – a systematic review

Nicolas Waespe, Sven Strebel, Simona Jurkovic Mlakar, Maja Krajinovic, Claudia Elisabeth Kuehni, Tiago Nava, and Marc Ansari.

Supplementary information A03

Table S03. Quality assessment tool: Detailed description for scoring (0-12 possible points; based on STREGA checklist[1] and adapted from Zazuli et al. and Leusink et al.).[2,3]

No.	Quality aspect	Points
1	**Quality of clinical information**	
	a. 1 point: mention the type of HSCT (allo/ auto) and the conditioning regimen	
	b. 1 point: adequate [description of] selection of participants with inclusion criteria, SOS criteria or objective lab parameters and baseline characteristics	
2	**Quality of genotyping**	
	a. 1 point: consideration of the Hardy–Weinberg equilibrium	
	b. 1 point: consideration of genotyping quality, for example, by reporting percentage of successful genotyping attempts or cross validation with a different technique	
3	**Quality in reporting of study population origin**	
	a. 1 point: mention geographical point of sample collection	
	b. 1 point: stratification or exclusion based on ethnicity, or statistical correction for population origin	
4	**Quality of sample size estimation and statistical correction for multiple testing**	
	a. 1 point: describe the power analysis to determined sample size	
	b. 1 point: any correction for multiple testing (e.g. Bonferroni, FDR)	
5	**Quality of study setup and analysis**	
	a. 1 point: description of possible variables that might affect outcome (e.g. treatment characteristics, patient risk factors, etc.)	
	b. 1 point: genotype-phenotype association adjusted for differences in treatments, patient risk factors etc. (multivariable analysis)	
6	**Replication of identified variants**	
	a. 1 point: replication of genetic variant associations in an independent cohort	
	b. 1 point: independent cohort is comparable to discovery cohort OR adequate correction for differences (multivariable analysis including variables associated with major differences int he discovery and replication cohort)	
	**Total points**	**(0 – 12)**

Table S04. Quality assessment of 27 included studies on genetic predictors for sinusoidal obstruction syndrome after HSCT or chemotherapy; after agreement was reached between authors.

Study author, journal, year	Clinical information		Genotyping		Study population origin		Sample size and statistical correction for multiple testing		Setup and analysis		Replication		Total points (agreement betweeen reviewers)
	(a)	(b)	(a)	(b)	(a)	(b)	(a)	(b)	(a)	(b)	(a)	(b)	
Duggan C, et al. Bone Marrow Transplant. 1999.[1]	1	1	0	0	1	0	0	0	0	0	0	0	3
Aplenc R, et al. Acta Haematologica. 2003[2]	1	1	0	0	1	0	0	0	0	0	0	0	3
Pihusch M, et al. Transplantation. 2004[3]	1	1	0	0	1	0	0	0	0	0	0	0	3
Srivastava A, et al. Blood. 2004[4]	1	1	0	0	1	0	0	0	1	1	0	0	5
Kallianpur AR et al. Bone Marrow Transplant. 2005[5]	1	1	1	1	1	1	0	0	1	1	0	0	**8**
Lennard L, et al. Clin. Pharmacol. Ther. 2006[6]	1	1	0	0	1	0	0	0	1	0	0	0	4
Elmaagacli AH, et al. Bone Marrow Transplant. 2007[7]	1	1	0	0	1	0	0	0	0	0	0	0	3
Goekkurt E, et al. Anticancer Res. 2007[8]	1	1	1	1	1	0	0	0	1	1	0	0	**7**
Kim I, et al. Annals of Hematol. 2007[9]	1	1	0	1	1	0	0	0	1	0	0	0	5
Lee KH, et al. Haematologica. 2007[10]	1	1	1	0	1	0	0	0	1	1	0	0	**6**
Zwaveling J, et al. Therapeut Drug Monitor. 2008[11]	1	1	1	0	1	0	0	0	0	0	0	0	4
Johnson L, et al. J Clin Pharmacol. 2008[12]	1	1	1	1	1	0	0	0	0	0	0	0	5
Rocha V, et al. Leukemia. 2009[13]	1	1	0	0	1	0	0	0	1	1	0	0	5
Elbahlawan L, et al. J Ped Hem Oncol. 2012[15]	1	1	1	1	1	0	0	0	0	0	0	0	5
Sucak GT, et al. Ann Hematology. 2012[16]	1	0	0	0	1	0	0	0	0	0	0	0	2
Krivoy N, et al. Curr Drug Safety. 2012[17]	1	1	0	0	1	0	0	0	0	0	0	0	3
Uppugunduri CRS, et al. Pharmacogenom J.2014[19]	1	1	1	0	1	0	0	0	0	0	0	0	4
Vreuls CPH, et al. Brit J Cancer. 2013[20]	1	1	0	0	1	0	0	0	1	1	0	0	5
Efrati E, et al. Bone Marrow Transplant. 2014[21]	1	0	0	0	1	0	0	0	0	0	0	0	2
Wray L, et al. Pediatr Blood Cancer. 2014[22]	1	1	1	1	1	1	1	0	1	1	0	0	**9**
Seifert C, et al. J. Cancer Res. Clin. Oncol. 2015.[23]	1	1	0	0	1	0	0	0	1	1	0	0	5
Ansari M, et al. Bone Marrow Transplant. 2016[24]	1	1	0	0	1	0	0	0	1	0	0	0	4
Byun JM, et al. PloS One. 2016[25]	1	1	0	0	1	0	0	0	1	1	0	0	5
Huezo-Diaz Curtis P, et al. Pharmacogenomics J. 2016[27]	1	1	1	0	1	0	1	0	0	0	0	0	5
Ansari M, et al. Oncotarget. 2017[28]	1	1	1	0	1	0	0	0	1	1	0	0	**6**
Ansari M, et al. Biol Blood Marrow Transpl. 2020[29,†]	1	1	1	0	1	0	0	1	1	1	1	0	**8**
Terakura S, et al. Int J Hematol. 2020[31]	1	1	0	0	1	0	0	0	1	0	0	0	4

Legend: †, includes populations described in Ansari M, et al. Bone Marrow Transplant. 2010[14] and Ansari M, et al. Bone Marrow Transplant. 2013;[18] Green shading: SOS after antineoplastic agent exposure.

References

1. Little J, Higgins JPT, Ioannidis JPA, et al. Strengthening the reporting of genetic association studies (STREGA): an extension of the strengthening the reporting of observational studies in epidemiology (STROBE) statement. *J Clin Epidemiol*. 2009;62(6):597-608.e4.
2. Zazuli Z, Vijverberg S, Slob E, et al. Genetic Variations and Cisplatin Nephrotoxicity: A Systematic Review. *Front Pharmacol*. 2018;9:1111.
3. Leusink M, Onland-Moret NC, de Bakker PI, de Boer A, Maitland-van der Zee AH. Seventeen years of statin pharmacogenetics: a systematic review. *Pharmacogenomics*. 2015;17(2):163–180.
4. Duggan C, Schmidt M, Lawler M, et al. The prothrombin gene variant G20210A but not factor V leiden may be associated with veno-occlusive disease following BMT. *Bone Marrow Transplant.* 1999;24(6):693–694.
5. Aplenc R, Vachani A, Han P, Glatfelter W, Sievers EL. Pharmacogenetics of gemtuzumab-associated hepatic sinusoidal occlusion syndrome after hematopoietic stem cell transplant. *Acta Haematol*. 2003;110(4):207–210.
6. Pihusch M, Lohse P, Reitberger J, et al. Impact of thrombophilic gene mutations and graft-versus-host disease on thromboembolic complications after allogeneic hematopoietic stem-cell transplantation. *Transplantation*. 2004;78(6):911–918.
7. Srivastava A, Poonkuzhali B, Shaji RV, et al. Glutathione S-transferase M1 polymorphism: a risk factor for hepatic venoocclusive disease in bone marrow transplantation. *Blood*. 2004;104(5):1574–1577.
8. Kallianpur AR, Hall LD, Yadav M, et al. The hemochromatosis C282Y allele: a risk factor for hepatic veno-occlusive disease after hematopoietic stem cell transplantation. *Bone Marrow Transplant.* 2005;35(12):1155–1164.
9. Lennard L, Richards S, Cartwright CS, et al. The thiopurine methyltransferase genetic polymorphism is associated with thioguanine-related veno-occlusive disease of the liver in children with acute lymphoblastic leukemia. *Clin. Pharmacol. Ther.* 2006;80(4):375–383.
10. Elmaagacli AH, Koldehoff M, Steckel NK, et al. Cytochrome P 450 2C19 loss-of-function polymorphism is associated with an increased treatment-related mortality in patients undergoing allogeneic transplantation. *Bone Marrow Transplantation*. 2007;40(7):659–664.
11. Goekkurt E, Stoehlmacher J, Stueber C, et al. Pharmacogenetic Analysis of Liver Toxicity after Busulfan/Cyclophosphamide-based Allogeneic Hematopoietic Stem Cell Transplantation. *Anticancer Res*. 2007;27(6C):4377–4380.
12. Kim I, Lee K-H, Kim JH, et al. Polymorphisms of the methylenetetrahydrofolate reductase gene and clinical outcomes in HLA-matched sibling allogeneic hematopoietic stem cell transplantation. *Ann Hematol*. 2007;86(1):41–48.
13. Lee K-H, Park SS, Kim I, et al. P2X7 receptor polymorphism and clinical outcomes in HLA-matched sibling allogeneic hematopoietic stem cell transplantation. *Haematologica*. 2007;92(5):651–657.
14. Zwaveling J, Press RR, Bredius RGM, et al. Glutathione S-transferase polymorphisms are not associated with population pharmacokinetic parameters of busulfan in pediatric patients. *Ther Drug Monit*. 2008;30(4):504–510.
15. Johnson L, Orchard PJ, Baker KS, et al. Glutathione S-transferase A1 genetic variants reduce busulfan clearance in children undergoing hematopoietic cell transplantation. *J Clin Pharmacol*. 2008;48(9):1052–1062.
16. Rocha V, Porcher R, Fernandes JF, et al. Association of drug metabolism gene polymorphisms with toxicities, graft-versus-host disease and survival after HLA-identical sibling hematopoietic stem cell transplantation for patients with leukemia. *Leukemia*. 2009;23(3):545–556.
17. Elbahlawan L, McArthur J, Quasney MW, et al. Association of IL-1β -511 polymorphism with severe veno-occlusive disease in pediatric-matched allogeneic hematopoietic stem cell transplantation. *J Pediatr Hematol Oncol*. 2012;34(3):175–179.
18. Sucak GT, Yaşar DG, Yegin ZA, et al. The prognostic role of hemochromatosis H63D allele in allogeneic hematopoietic stem cell transplantation. *Ann Hematol*. 2012;91(8):1281–1287.

19. Krivoy N, Zuckerman T, Elkin H, et al. Pharmacokinetic and pharmacogenetic analysis of oral busulfan in stem cell transplantation: prediction of poor drug metabolism to prevent drug toxicity. *Curr Drug Saf*. 2012;7(3):211–217.
20. Uppugunduri CRS, Rezgui MA, Diaz PH, et al. The association of cytochrome P450 genetic polymorphisms with sulfolane formation and the efficacy of a busulfan-based conditioning regimen in pediatric patients undergoing hematopoietic stem cell transplantation. *Pharmacogenomics J.* 2014;14(3):263–271.
21. Vreuls CPH, Olde Damink SWM, Koek GH, et al. Glutathione S-transferase M1-null genotype as risk factor for SOS in oxaliplatin-treated patients with metastatic colorectal cancer. *Br. J. Cancer*. 2013;108(3):676–680.
22. Efrati E, Zuckerman T, Ben-Ami E, Krivoy N. MTHFR C677T/A1298C genotype: a possible risk factor for liver sinusoidal obstruction syndrome. *Bone Marrow Transplant.* 2014;49(5):726–727.
23. Wray L, Vujkovic M, McWilliams T, et al. TPMT and MTHFR genotype is not associated with altered risk of thioguanine-related sinusoidal obstruction syndrome in pediatric acute lymphoblastic leukemia: a report from the Children's Oncology Group. *Pediatr Blood Cancer*. 2014;61(11):2086–2088.
24. Seifert C, Wittig S, Arndt C, Gruhn B. Heparanase polymorphisms: influence on incidence of hepatic sinusoidal obstruction syndrome in children undergoing allogeneic hematopoietic stem cell transplantation. *J. Cancer Res. Clin. Oncol.* 2015;141(5):877–885.
25. Ansari M, Huezo-Diaz P, Rezgui MA, et al. Influence of glutathione S-transferase gene polymorphisms on busulfan pharmacokinetics and outcome of hematopoietic stem-cell transplantation in thalassemia pediatric patients. *Bone Marrow Transplant*. 2016;51(3):377–383.
26. Byun JM, Kim H-L, Shin D-Y, et al. The Impact of Methylenetetrahydrofolate Reductase C677T Polymorphism on Patients Undergoing Allogeneic Hematopoietic Stem Cell Transplantation with Methotrexate Prophylaxis. *PLoS One*. 2016;11(10):e0163998.
27. Huezo-Diaz Curtis P, Uppugunduri CRS, Muthukumaran J, et al. Association of CTH variant with sinusoidal obstruction syndrome in children receiving intravenous busulfan and cyclophosphamide before hematopoietic stem cell transplantation. *Pharmacogenomics J.* 2016;
28. Ansari M, Curtis PH-D, Uppugunduri CRS, et al. GSTA1 diplotypes affect busulfan clearance and toxicity in children undergoing allogeneic hematopoietic stem cell transplantation: a multicenter study. *Oncotarget*. 2017;8(53):90852–90867.
29. Ansari M, Petrykey K, Rezgui MA, et al. Genetic Susceptibility to Hepatic Sinusoidal Obstruction Syndrome in Pediatric Patients Undergoing Hematopoietic Stem Cell Transplantation. *Biol. Blood Marrow Transplant.* 2020;26(5):920–927.
30. Terakura S, Onizuka M, Fukumoto M, et al. Analysis of glutathione S-transferase and cytochrome P450 gene polymorphism in recipients of dose-adjusted busulfan-cyclophosphamide conditioning. *Int J Hematol*. 2020;111(1):84–92.
