# Supplementary material for: Genetic Predictors for Sinusoidal Obstruction Syndrome—A Systematic Review
Source: J Pers Med. 2021 Apr 26;11(5):347. doi: 10.3390/jpm11050347 (PMC8145271; doi:10.3390/jpm11050347)
Supplement: Supplementary file 1 [file jpm-11-00347-s001.zip › A02_Waespe_SOS PGX Review_Supplement_search_FINAL.pdf]

Genetic predictors for sinusoidal obstruction syndrome – a systematic review

Nicolas Waespe, Sven Strebel, Simona Jurkovic Mlakar, Maja Krajinovic, Claudia Elisabeth Kuehni, Tiago Nava, and Marc Ansari.

Supplementary information A02

Table S02. Detailed search strategy to identify manuscripts of genetic risk analysis with sinusoidal obstruction syndrome (PubMed).

Population: HSCT, antineoplastic agents
("Stem Cell Transplantation"[Mesh] OR "Hematopoietic Stem Cell Transplantation"[Mesh] OR "Peripheral Blood Stem Cell Transplantation"[Mesh] OR "Bone Marrow Transplantation"[Mesh] OR "Transplantation Conditioning"[Mesh] OR (hematopoietic stem cell transplant*[tiab] OR haematopoietic stem cell transplant*[tiab] OR hematopoietic cell transplant*[tiab] OR haematopoietic cell transplant*[tiab] OR stem cell transplant*[tiab] OR HSC transplant*[tiab] OR peripheral blood stem cell transplant*[tiab] OR peripheral stem cell transplant*[tiab] OR peripheral blood cell transplant*[tiab] OR peripheral blood progenitor cell transplant*[tiab] OR progenitor cell transplant*[tiab] OR stem cell*[tiab] OR bone marrow transplant*[tiab] OR marrow transplant*[tiab] OR blood transplant*[tiab] OR hematopoietic stem cell mobilization[tiab] OR hematopoietic stem cell mobilisation[tiab] OR haematopoietic stem cell mobilization[tiab] OR haematopoietic stem cell mobilisation[tiab] OR allograft*[tiab] OR autograft* OR homograft* OR ((allogen*[tiab] OR allogeneic[tiab] OR autolog*[tiab] OR homolog*[tiab]) AND (transplant*[tiab] OR graft*[tiab] OR rescue*[tiab])) OR stem cell rescue*[tiab] OR bone marrow rescue*[tiab] OR bone marrow graft*[tiab] OR bone marrow cell transplant*[tiab] OR cord blood stem cell transplant*[tiab] OR placental blood stem cell transplant*[tiab] OR umbilical cord stem cell transplant*[tiab] OR conditioning regimen*[tiab] OR ABMT[tiab] OR ASCT[tiab] OR BMT[tiab] OR PBPC[tiab] OR PBSCT[tiab] OR PSCT[tiab] OR SCT[tiab] OR HSCT[tiab]))<br><br>**OR**<br><br>("Antineoplastic Agents"[Mesh] OR "Antineoplastic Agents" [Pharmacological Action] OR "Antimetabolites, Antineoplastic" [Pharmacological Action] OR "Antineoplastic Protocols"[Mesh] OR "Chemotherapy, Adjuvant"[Mesh] OR "Neoadjuvant Therapy"[Mesh] OR "Chemoradiotherapy"[Mesh] OR "Chemoradiotherapy, Adjuvant"[Mesh] OR "Consolidation Chemotherapy"[Mesh] OR "Induction Chemotherapy"[Mesh] OR "Maintenance Chemotherapy"[Mesh] OR (antineoplastic*[tiab] OR anti-neoplastic*[tiab] OR antitumour*[tiab] OR anti-tumour*[tiab] OR antitumor*[tiab] OR anti-tumor*[tiab] OR anti-cancer*[tiab] OR anticancer*[tiab] OR anticarcinogen*[tiab] OR anti-carcinogen*[tiab] OR maintain[tiab] or maintenance[tiab] or consolidat*[tiab] OR chemotherap*[tiab]))
Exposure: Genetic variants
("Genetic Variation"[Mesh] OR "Polymorphism, Genetic"[Mesh] OR "Genotype"[Mesh] OR "Genetics"[Mesh] OR "Pharmacogenetics"[Mesh] OR "Polymorphism, Genetic"[Mesh] OR "Genetic Predisposition to Disease"[Mesh] OR "genetics" [Subheading] OR (genetic*[tiab] OR pharmacogen*[tiab] OR pharmaco-gen*[tiab] OR genotyp*[tiab] OR gene[tiab] OR genes[tiab] OR genom*[tiab] OR polymorphism*[tiab] OR predispos*[tiab] OR susceptib*[tiab] OR inherit*[tiab] OR heredit*[tiab] OR heritable[tiab] OR familial[tiab] OR variant*[tiab] OR mutation*[tiab]))
Outcome: Sinusoidal obstruction syndrome
("Hepatic Veno-Occlusive Disease"[Mesh] OR (veno-occlusive disease*[tiab] OR veno-oclusive disease*[tiab] OR venoocclusive disease*[tiab] OR venooclusive disease*[tiab] OR veno-occlusive liver disease*[tiab] OR veno-occlusive hepatic disease*[tiab] OR venoocclusive liver disease*[tiab] OR venoocclusive hepatic disease*[tiab] OR venocclusive liver disease*[tiab] OR sinusoidal obstruction syndrome*[tiab] OR sinusoidal obstructive syndrome*[tiab] OR sinusoidal obstruction*[tiab] OR stuart bras[tiab] OR VOD[tiab] OR HVOD[tiab] OR VOD-SOS[tiab] OR SOS-VOD[tiab] OR HPVOD[tiab]))
**Limits**
NOT (animals[mh] NOT humans[mh]) NOT (letter[pt] OR news[pt] OR comment[pt] OR editorial[pt] OR congress[pt] OR abstracts[pt])
